# Association between sarcopenia with lifestyle and family function among community-dwelling Chinese aged 60 years and older

**DOI:** 10.1186/s12877-017-0587-0

**Published:** 2017-08-18

**Authors:** Shan Hai, Hui Wang, Li Cao, Ping Liu, Jianghua Zhou, Ying Yang, Birong Dong

**Affiliations:** Department of Geriatrics, West China Hospital, Sichuan University, 37 Guoxue Lane, Chengdu, NO 610041 China

**Keywords:** Chinese, Community, Family APGAR scale, Sarcopenia

## Abstract

**Background:**

Sarcopenia is defined as the age-related decline in skeletal muscle mass and function. The risk factors and causes of sarcopenia must be identified to develop prevention and treatment strategies for this syndrome. Our aim was to examine the association between sarcopenia with lifestyle and family function among community-dwelling Chinese people aged 60 years and older.

**Methods:**

We conducted this study to evaluate sarcopenia among 834 community-dwelling Chinese individuals aged ≥60 years using the Asian Working Group for Sarcopenia (AWGS) criteria. The sociodemographic characteristics, food consumption patterns, habits of smoking, and alcohol consumption of the participants were collected using a general questionnaire, whereas physical activity was assessed using the International Physical Activity Questionnaire (IPAQ; long-form version). Family function was assessed using the Family APGAR scale. In addition, the association of sarcopenia with lifestyle and family function was examined using univariate and multivariate analyses.

**Results:**

The total prevalence rate of sarcopenia was 10.6%. Female participants with sarcopenia had a lower frequency per week of nut consumption than those without sarcopenia (*p* < 0.05), whereas, for male participants, the differences in food consumption patterns of those with sarcopenia versus those without sarcopenia were not significant. Among the participants, the mean Family APGAR score was 8 (standard deviation [SD] = 0.92). For both sexes, participants with sarcopenia had lower family function scores than those without sarcopenia. In the multivariate model, after adjustment for all covariates, frequency per week of nut consumption (adjusted OR 0.724, 95% CI 0.532–0.985, *P* < 0.05) and Family APGAR score (adjusted OR 0.683, 95% CI 0.496–0.940, *P* = 0.019) were statistically significantly associated with sarcopenia. The relationship between other lifestyle habits and sarcopenia was not significant.

**Conclusion:**

There was significant association between sarcopenia with intake of nuts and family function. Further studies should evaluate if adequate intake of nuts and a well-functioning family may be effective in lowering the risk of sarcopenia.

## Background

Sarcopenia is defined as the age-related decline in skeletal muscle mass and function [1], which may result in falls, physical disabilities, fracture, loss of dependence, poor quality of life and increased risk of death in older people [2]. Sarcopenia is also a common health problem that is associated with a high personal consumption and increased medical costs. The health care cost of sarcopenia in the United States alone was estimated at 18.5 billion dollars in the year of 2000 [3]. Therefore, reducing the incidence of sarcopenia and improving health-related quality of life in patients with sarcopenia are becoming increasingly important areas of research.

The risk factors and causes of sarcopenia must be identified to develop prevention and treatment strategies for this disease, particularly concerning lifestyle habits, which are more controllable in comparison to age-related systemic changes and genetic factors. According to several recently published studies, a diet characterized by an adequate intake of protein, a high intake of essential amino acids (EAA), particularly leucine-rich food such as beef, fish, and legumes and supplementation of vitamin D, is associated with a reduced risk for developing sarcopenia [4–6]. However, other lifestyle habits such as chronic alcohol consumption and smoking may promote loss of muscle mass and strength in old age indicating that they may be risk factors for sarcopenia [7, 8]. Some cross-sectional and prospective studies have demonstrated that higher levels of physical activity have positive effects on muscle mass or strength [9, 10]. However, findings from other studies have conflicted regarding whether or not lower self-reported physical activity is associated with a greater likelihood of developing sarcopenia [11]. Currently, a majority of studies on the association between sarcopenia and lifestyle are from Western countries. However, limited studies have reported on the prevalence and risk factors of sarcopenia in Chinese adults, particularly in community-dwelling individuals.

Family function has been found to be associated with health conditions of the elderly in several studies [12, 13]. For example, many elderly adults with chronic health conditions have severe family dysfunction, and poor family function scores are correlated with the presence of chronic illness [14]. For most elderly Chinese adults, families are their primary source of social support, which is a recognized determinant of health. However, presently, fewer studies have focused on the relationship between sarcopenia and family function.

Because genetic background, ethnicity, and living environment differ from other countries among the Chinese population, the precise factors associated with sarcopenia must be defined. Thus, the aim of this study was to determine the association of sarcopenia with lifestyle and family function among the community-dwelling population aged ≥60 years.

## Methods

### Study design

We used various advertising strategies mostly including displaying posters to recruit volunteers in the Wuhou District, Chengdu, Sichuan province in southwest China on convenience sampling basis. Individuals aged ≥60 years who lived in the community for more than 12 months were recruited for the study. Participants with the following conditions were excluded from the study: (1) physical disabilities such as loss of hand or foot or limbs; (2) an implanted electronic device or orthopedic metal implantations. (3) unable to perform the handgrip strength test or walking test;(4)dementia and therefore, unable to communicate with interviewers; or (5) later stages of malignancies with cachexia; A total of 848 persons aged ≥60 years were recruited from the community population of Chengdu from August 2014 to July 2015. Of these participants, 14 were excluded from the study because of missing data. We collected and analyzed data for 834 participants (415 men and 419 women).

Trained personnel visited all the participants in person and collected the data. Sociodemographic characteristics including educational level, age, sex and occupation were recorded using a general questionnaire. Cognitive function was measured with the 30-item MMSE, a global test with the following components: attention, orientation, language, calculation, and recall. (sensitivity 80–90%, specificity 80–100%) [15]. We assessed depression using short form 15-items Chinese version of Geriatric Depression Scale (GDS-15) [16]. The prevalence of specific medical conditions was established using standardized criteria that combined the history of the physical illness, diagnosis, and corresponding medication. The anthropometric variables of all participants were checked. Body weight was measured in light clothing to the nearest 0.01 kg, and height was measured to the nearest 0.01 cm. Body mass index (BMI) was calculated as weight in kilograms divided by height squared in meters (kg/m^2^).

### Collection of information on lifestyle

We collected data on lifestyle. Lifestyle criteria included dietary patterns, physical activity, and smoking and drinking habits. A validated simplified FFQ was used for assessing dietary information. All participants completed the questionnaire about the frequency of eating each food item and the frequency units (day, week, month, or never) based on nine food categories: grain (or cereals), vegetables, fruit, meat (pork, beef, poultry, and mutton), eggs, fish and shrimp, milk and milk products, legumes and nuts. The nine kinds of foods were based on the Chinese Food Guide Pagoda, which is extensively used for the dietary guidelines of Chinese residents. Each food category frequency was converted into times per week (times/week). The validity and reliability of FFQ have been described elsewhere [17]. Participants were classified as current smokers, past smokers and never smoker. Subjects who had smoked everyday in the past year were classified as current smokers, whereas those who had smoked every day for more than 2 years in the year before the study began were classified as past smokers. They were also classified by their consumption of alcohol into no-drinkers, low-risk drinkers, and high-risk drinkers. High-risk drinking was defined as drinking more than twice a week. Those who consumed alcohol less than twice a week were classified as low-risk drinkers. The questionnaire had yes or no items only. Physical activity was assessed using a Chinese version of the International Physical Activity Questionnaire (IPAQ; long form) [18]. 

### Assessment of family function

Family function was assessed using the Family APGAR score. The Family APGAR scale, designed by Smilkstein in 1978, is used to evaluate an individual’s satisfaction with their perceived level of family function [19]. It consists of five parameters: Adaptability, Partnership, Growth, Affection, and Resolve. A three-point scale ranging from 0 (hardly ever) to 2 (almost always) is used to score all the items. The total score range varies from 0 to 10. Higher scores indicate higher levels of satisfaction with family functioning. A score of 0–3 indicates severe family dysfunction, 4–7 moderate family dysfunction, and 8–10 good family function. It has been used widely in China, with satisfactory validity and reliability [20, 21]. 

### Assessment of sarcopenia

We defined sarcopenia, according to the AWGS criteria, [2] as the presence of low muscle mass, plus low muscle strength, or low physical performance.

Muscle mass was measured using bioelectrical impedance analysis (BIA) (Maltron BioScan 920 II, Rayleigh, UK). Appendicular skeletal muscle mass (ASM) was calculated as the sum of skeletal muscle in the arms and legs. The height-adjusted appendicular skeletal muscle mass (ASMI) was defined as ASM divided by height squared in meters (ASM/height^2^). Low muscle mass was defined as a height-adjusted ASM of less than 7.0 kg/m^2^ in men and 5.7 kg/m^2^ in women [2]. Muscle strength was assessed by grip strength and measured using a dynamometer (CAMRYEH 101, China). Participants exert maximum effort three times with each hand. Three trials for each hand were carried out and we used the highest value for diagnosing sarcopenia. Low handgrip strength was defined as <26 kg in men and <18 kg in women, using the AWGS consensus cutoff points [2]. Usual gait speed (m/s) on a 6 m course was used as an objective measure of physical performance and a slow walking speed was defined as a walking speed less than 0.8 m/s [2].

### Statistical analysis

SPSS21.0 was used to perform statistical analyses. Continuous variables were presented as mean ± standard deviation (M ± SD). Significance testing of the difference between the two groups was conducted using the independent samples t-test for continuous variables and the chi-square test or Fisher’s exact test for categorical variables. Multiple logistic regression analysis was used to evaluate the association between risk factors and sarcopenia. A two-sided *p* < 0.05 was considered statistically significant.

## Results

Data from 834 participants were used for the analysis. The mean age was 68.57 ± 6.44 years (range: 60–92 years) and 419(50.2%) participants were female. Of the 834 community-dwelling elderly Chinese people, 88(10.6%) participants were classified as having sarcopenia. For male, 23(48.9%) of the participants with sarcopenia had low ASMI and low grip strength, 17(36.2%) had low ASMI and low usual gait speed, 7(14.9%) had both low muscle strength and low physical performance. These rates were 22(53.7%), 19(46.3%) and 0(0%) in women, respectively.

Table 1 presents the socioeconomic status and clinical characteristics of the participants by sex and sarcopenia status. Older participants were more likely to have sarcopenia than younger ones (*p* < 0.001). Participants with sarcopenia had statistically significantly lower BMI, ASM, ASMI, handgrip strength, and usual walking speed than participants without sarcopenia. History of cardiac diseases, hypertension, and stroke was more common in sarcopenic men than in nonsarcopenic participants.

**Table 1:** Baseline characteristics profile of participants

	Male(*n* = 415)			Female(*n* = 419)		
	Sarcopenia *N* = 47	Non-sarcopenia *N* = 368	*P*	Sarcopenia *N* = 41	Non-sarcopenia *N* = 378	*P*
Age, yrs., Mean(SD)	73.64(7.25)	68.28(6.24)	<0.001**	72.85(6.98)	67.76(6.03)	<0.001**
Weight, Kg, Mean(SD)	59.01(10.75)	64.92(9.00)	<0.001**	51.61(9.49)	56.82(8.34)	<0.001**
Height,cm, Mean(SD)	160.19(5.31)	164.50(6.04)	<0.001**	151.22(6.09)	152.99(5.96)	0.071
BMI,kg/m2, Mean(SD)	22.99(3.87)	23.96(2.76)	0.031*	22.52(3.71)	24.26(3.15)	0.001**
Appendicular muscle mass, kg, Mean(SD)	15.41(2.07)	17.29(2.55)	<0.001**	11.64(1.17)	13.33(1.98)	<0.001**
ASMI, kg/m2, Mean(SD)	5.99(0.63)	6.38(0.80)	<0.001**	5.09(0.46)	5.69(0.72)	<0.001**
Grip strength, kg, Mean(SD)	25.99(5.21)	36.09(6.29)	<0.001**	17.53(3.19)	22.99(4.46)	<0.001**
Usual gait speed, m/s, Mean(SD)	0.86(0.26)	1.09(0.17)	<0.001**	0.85(0.18)	1.03(0.15)	<0.001**
Education years, n(%)						
0	5(10.6)	5(1.4)	<0.001**	6(14.6)	9(2.4)	<0.001**
1–6	28(59.6)	156(42.4)		25(61.0)	145(38.4)	
7–9	6(12.8)	49(13.3)		5(12.2)	57(15.1)	
10–12	7(14.9)	153(41.6)		5(12.2)	159(42.1)	
>12	1(2.1)	5(1.4)		0	8(2.1)	
Diseases, n(%)						
Cardiac diseases	5(10.6)	14(3.8)	0.035*	1(2.4)	17(4.5)	0.537
Kidney disease	1(2.1)	4(1.1)	0.538	0(0.0)	7(1.9)	0.380
Hypertension	26(55.3)	144(39.1)	0.034*	21(51.2)	156(41.3)	0.221
Stroke history	2(4.3)	2(0.5)	0.014*	0(0.0)	4(1.1)	0.508
Cancer	0(0.0)	2(0.5)	0.612	0(0.0)	2(0.1)	0.541
Diabetes	11(23.4)	59(16.0)	0.204	12(29.3)	65(17.2)	0.058
COPD	1(2.1)	3(0.8)	0.386	0(0.0)	7(1.9)	0.380
MMSE scores	26.6(1.9)	27.1(2.4)	0.079	25.4(4.0)	26.3(3.2)	0.088
GDS-15 scores	1.6(1.3)	1.5(1.7)	0.780	1.8(1.9)	1.5(1.9)	0.414

* *p* < 0.05; **p < 0.01 between non sarcopenic and sarcopenic individuals;BMI = body mass index; ASMI = appendicular skeletal muscle mass index; COPD = chronic obstructive pulmonary disease. Using independent-samples *t* test for continuous variables and Pearson chi-square or Fisher’s exact test

(where an expected cell count was < 5) for categorical variables. During testing, *P* < 0.05 was considered statistically significant

Table 2 presents the lifestyle and family function score of participants by sex and sarcopenia status. Female participants who did not drink alcohol were more common in the sarcopenia group (*p* < 0.05). For men, the level of physical activity was significantly different between participants with or those without sarcopenia. Female participants with sarcopenia had statistically significantly lower frequency per week of nut consumption than those without sarcopenia. However, no statistically significant difference in regard to food items was observed between men with and those without sarcopenia. The mean Family APGAR score was 8 (SD = 0.92). For both sexes, participants with sarcopenia had lower family function scores than those without sarcopenia. The prevalence rate of different levels of family function was significantly different in participants with or without sarcopenia for both sexes.

**Table 2:** Lifestyle and family function of participants

	Male(*n* = 415)			Female(*n* = 419)		
	Sarcopenia *N* = 47	Non-sarcopenia *N* = 368	*P*	Sarcopenia *N* = 41	Non-sarcopenia *N* = 378	*P*
Smoking, n(%)						
Current Smokers	14(29.8)	106(28.8)	0.413	0(0.0)	9(2.4)	0.445
Quitter	15(31.9)	151(41.0)		2(4.9)	10(2.6)	
Never Smokers	18(38.3)	111(30.2)		39(95.1)	359(95.0)	
Drinking, n(%)						
Not drink	32(68.1)	204(55.4)	0.252	41(100.0)	327(86.5)	0.046*
Drinking <2/week	6(12.8)	61(16.6)		0(0.0)	21(5.6)	
Drinking ≥2/week	9(19.1)	103(28.0)		0(0.0)	30(7.9)	
Present Physical activity, n(%)						
Low	4(8.5)	7(1.9)	0.020*	1(2.4)	6(1.6)	0.387
Moderate	20(42.6)	141(38.4)		14(34.1)	97(25.7)	
High	23(48.9)	219(59.7)		25(61.0)	275(72.7)	
Dietary Pattern(times per week, Mean(SD))						
Grain,Cereals	20.00(3.95)	20.21(3.79)	0.726	20.00(3.67)	19.37(3.85)	0.315
Vegetables	14.98(12.61)	17.12(11.66)	0.242	18.22(11.11)	16.90(11.92)	0.498
Fruits	5.11(3.90)	5.22(3.71)	0.840	5.39(3.29)	6.44(3.53)	0.071
Eggs	5.36(3.02)	4.86(2.89)	0.263	4.32(2.62)	4.73(2.72)	0.355
Fish, shrimp	1.04(1.49)	0.85(1.29)	0.353	0.98(1.54)	0.90(1.30)	0.744
Nuts	0.19(0.74)	0.61(1.90)	0.135	0.05(0.22)	0.81(2.11)	0.022*
Meat(pork,beef,mutton,poultry)	8.87(4.79)	9.54(5.01)	0.386	9.54(4.72)	9.15(4.50)	0.604
Milk,milk products	4.87(3.40)	4.45(4.45)	0.532	3.86(3.14)	4.68(3.17)	0.112
legumes	3.02(4.26)	2.28(2.57)	0.090	2.17(3.32)	2.23(2.61)	0.888
Family function						
APGAR,Mean(SD)	7.68(1.11)	8.05(0.77)	0.003**	7.63(1.22)	8.06(0.99)	0.013*
Good family function	40(85.1)	355(96.5)	0.001**	35(85.4)	363(96.0)	0.004**
Moderate family dysfunction	7(14.9)	12(3.2)		5(12.2)	9(2.4)	
severe family dysfunction	0(0.0)	1(0.3)		1(2.4)	6(1.6)	
Live along, n(%)	4(8.5)	19(5.2)	0.345	8(19.5)	48(12.7)	0.088

* *p* < 0.05; ** *p* < 0.01 between non sarcopenic and sarcopenic individuals. During testing, *P* < 0.05 was considered statistically significant

The association of lifestyle with sarcopenia is shown in Table 3. After adjustment for sex, age, educational levels, diabetes, hypertension, heart disease and stroke, a significant association was revealed between prevalence of sarcopenia and frequency per week of nut consumption (0.724, 95% CI 0.532–0.985; *P* < 0.05). As for the other eight food items, no statistical significance was detected in either the unadjusted or adjusted models. In the unadjusted model, high level of physical activity (OR, 0.224; 95% CI, 0.074–0.672; *P* = 0.008) and drinking ≥ 2 times per week (OR, 0.435; 95% CI, 0.206–0.919; *P* = 0.029) was found to be associated with sarcopenia. However, the effect disappeared after adjusting for other covariates. Multiple logistic regression models were also used to examine the association of family function with sarcopenia in Table 3. There was a significant association of Family APGAR scores with sarcopenia (0.683, 95% CI 0.496–0.940, *P* = 0.019).

**Table 3:** Unadjusted and adjusted model for factors related to Sarcopenia

Factors	Unadjusted OR(95% CI)	Adjusted OR(95% CI)
Dietary pattern		
Grain, Cereals	1.015(0.956–1.078)	1.018(0.952–1.089)
Vegetables	0.998(0.979–1.017)	1.001(0.980–1.023)
Fruits	0.956(0.895–1.022)	0.961(0.888–1.039)
Eggs	1.007(0.932–1.088)	0.996(0.914–1.087)
Fish,shrimp	1.061(0.913–1.232)	1.012(0.866–1.181)
Nuts	0.702(0.516–0.956)^*^	0.724(0.532–0.985)^*^
Meat	0.993(0.946–1.042)	1.000(0.948–1.054)
Milk, milk products	0.990(0.932–1.052)	0.957(0.883–1.037)
Legumes	1.034(0.962–1.112)	1.016(0.941–1.098)
Physical activity		
Low	1(reference)	1(reference)
Moderate	0.337(0.111–1.026)	0.407(0.093–1.779)
High	0.224(0.074–0.672)^*^	0.372(0.085–1.634)
Smoking		
Never Smoker	1(reference)	1(reference)
Current smokers	1.137(0.630–2.053)	1.365(0.585–3.186)
quitter	0.773(0.426–1.403)	0.637(0.286–1.423)
Drinking		
Not drink	1(reference)	1(reference)
Drinking <2/week	0.530(0.218–1.294)	0.610(0.235–1.579)
Drinking ≥2/week	0.435(0.206–0.919)^*^	0.527(0.233–1.192)
APGAR	0.699(0.576–0.848)^*^	0.683(0.496–0.940)^*^

Binary logistic analyses were used to estimate the OR and 95% CI of factors related to sarcopenia; Adjusted for gender, ages, educational levels,diabetes, hypertension, heart disease, stroke, MMSE score and GDS score

*P < 0.05

## Discussion

The present study was performed to evaluate the prevalence of sarcopenia among Chinese community-dwelling people aged ≥60 years using the AWGS definition. Among the study population, 10.6% were diagnosed with sarcopenia. The prevalence of sarcopenia observed in this study was similar to the prevalence observed in Japanese and Korean populations, [22, 23] however, lower than that in Caucasian populations, [24, 25] This difference might partly originate from heterogeneity of study populations, but also due to the different techniques used to assess muscle mass.

Although sarcopenia can be explained by some fixed factors, many factors are not modifiable. This fact has led to increasing interest in the influence of lifestyle on muscle mass and function in older people. These factors include various aspects of food consumption patterns, physical activity, alcohol intake, and tobacco use. For older adults, altered taste and smell, social changes, and economic limitations may also lead to decreased food intake, [26] especially a low nutrient intake. Inadequate intake of nutrients is one of the major mechanisms underlying sarcopenia. Recent studies have demonstrated that an intake of nutrients, in particular, protein intake, EAA, and Vitamin D, have an influence on skeletal muscle metabolism [4–6]. In this study, after adjustment for sex, ages, educational levels, diabetes, hypertension, heart disease and stroke, the frequency per week of nut consumption was significantly associated with sarcopenia (0.724, 95% CI 0.532–0.985; *P* < 0.05). An adequate intake of nuts may be a protective factor against sarcopenia. Most of the participants in the study had a diet inclusive of nuts, including peanuts, walnut kernels or cashews, which abound in EAA such as leucine, valine, methionine and tryptophan. Recent studies have demonstrated that the leucine attenuated skeletal muscle wasting occurs by an interaction with proteolytic pathways [5]. Katsanos et al. has shown that increasing the proportion of leucine in a mixture of EAA given to elderly subjects can reverse the attenuated response of muscle protein synthesis [27]. An adequate intake of nuts that are abundant in leucine may serve as a potential strategy to combat the progression of sarcopenia. Our study provided supporting evidence about the association of the intake of nutrients and sarcopenia.

Chronic alcohol intake and current smoking are other lifestyle habits that have been associated with sarcopenia in previous studies [7, 8]. In the present study, after adjustment for sex, ages, educational levels, diabetes, hypertension, heart disease, and stroke, no significant difference was revealed between sarcopenia and these habits. This variance might be due to the type of questions regarding these habits on the questionnaire, which did not specify quantity or frequency of smoking or alcohol consumption, so we could not conclude whether or not there was an excess of alcohol and tobacco consumption in these participants. Therefore, the association between smoking and alcohol intake with sarcopenia should be further investigated. In the multivariate model, physical activity was not significantly associated with sarcopenia. Previous study showed resistance training was demonstrated to be more effective in attenuating the development of sarcopenia [9, 28]. The conflicting results may be explained for the different physical activities. Because in the study participants only took part in some baseline physical activities, which may be inactive compared with other strength training programs.

For most Chinese elderly, families are the primary source of social support, which is a recognized determinant of health [29]. Several studies found family function affected the health conditions and quality of the care and services for the aged with some chronic diseases [30]. If older adults enjoy a well-functioning family life or family support, their health functions will also enable them to enjoy good daily functioning and self-care ability [12]. However, presently few studies have focused on the relationship between family function and sarcopenia. To the best of our knowledge, this was the first research study that has focused on the association between family function and sarcopenia. In this study, after adjusting for age, sex, and education level, and medical disease, a significant association was found between family function score and sarcopenia. However, the latent basis of the association between family function and sarcopenia is uncertain. Family function is associated with psychological outcomes, metabolic control, and eating behaviors or dietary patterns in the elderly. These may be possible mechanisms to consider as an underlying basis of this association. Further studies should evaluate if good family function may be effective in lowering the risk of sarcopenia.

This study has several strengths. First, it is one of the studies in Asia to use the Asian Working Group for Sarcopenia criteria in Asia. Second, the study was conducted using a relatively large sample in China, which was a well-characterized population of community-dwelling older adults living in a defined geographical area. Our sample included participants that ranged in age from 60 to 92 years. There were several limitations of our study. First, the assessment of lifestyle and family function was based on cross-sectional measures, so it was not possible to determine causal relationships. Second, only ambulatory participants were surveyed because participants who were in hospitals or had serious diseases were not included.

## Conclusion

In conclusion, we found the prevalence of sarcopenia among community-dwelling Chinese population aged 60 years and older was high and there was significant association between sarcopenia and intake of nuts and family function.

## Abbreviations

ASM: Appendicular skeletal muscle mass
ASMI: Appendicular skeletal muscle mass index
AWGS: Asian Working Group for Sarcopenia
BIA: Bioelectrical impedance analysis
BMI: Body mass index
EWGSOP: European Working Group on Sarcopenia in Older People
GS: Gait speed
HS: Handgrip strength
